# A “Do-It-Yourself” phenotyping system: measuring growth and morphology throughout the diel cycle in rosette shaped plants

**DOI:** 10.1186/s13007-017-0247-6

**Published:** 2017-11-08

**Authors:** Andrei Dobrescu, Livia C. T. Scorza, Sotirios A. Tsaftaris, Alistair J. McCormick

**Affiliations:** 1 Institute of Digital Communications, School of Engineering, University of Edinburgh, Edinburgh, EH9 3FB UK; 2 Daniel Rutherford Building, SynthSys and Institute of Molecular Plant Sciences, School of Biological Sciences, University of Edinburgh, The King’s Buildings, Edinburgh, EH9 3BF UK

**Keywords:** 2D, *Arabidopsis thaliana*, Near-infrared LED array, Image analysis, Low cost, Rubisco

## Abstract

**Background:**

Improvements in high-throughput phenotyping technologies are rapidly expanding the scope and capacity of plant biology studies to measure growth traits. Nevertheless, the costs of commercial phenotyping equipment and infrastructure remain prohibitively expensive for wide-scale uptake, while academic solutions can require significant local expertise. Here we present a low-cost methodology for plant biologists to build their own phenotyping system for quantifying growth rates and phenotypic characteristics of *Arabidopsis thaliana* rosettes throughout the diel cycle.

**Results:**

We constructed an image capture system consisting of a near infra-red (NIR, 940 nm) LED panel with a mounted Raspberry Pi NoIR camera and developed a MatLab-based software module (iDIEL Plant) to characterise rosette expansion. Our software was able to accurately segment and characterise multiple rosettes within an image, regardless of plant arrangement or genotype, and batch process image sets. To further validate our system, wild-type Arabidopsis plants (Col-0) and two mutant lines with reduced Rubisco contents, pale leaves and slow growth phenotypes (*1a3b* and *1a2b*) were grown on a single plant tray. Plants were imaged from 9 to 24 days after germination every 20 min throughout the 24 h light–dark growth cycle (i.e. the diel cycle). The resulting dataset provided a dynamic and uninterrupted characterisation of differences in rosette growth and expansion rates over time for the three lines tested.

**Conclusion:**

Our methodology offers a straightforward solution for setting up automated, scalable and low-cost phenotyping facilities in a wide range of lab environments that could greatly increase the processing power and scalability of Arabidopsis soil growth experiments.

**Electronic supplementary material:**

The online version of this article (10.1186/s13007-017-0247-6) contains supplementary material, which is available to authorized users.

## Background

There is an urgent need for novel technologies that monitor and predict the impact of abiotic (e.g. light, temperature) and biotic (e.g. pests, disease) stresses on plant growth and productivity at a large scale. Understanding the dynamic interactions between genotype, phenotype and the growth environment are key to predicting plant performance, resource use efficiency, stress tolerance and yield (for review see [1, 2]). Although molecular profiling technologies now enable the generation of large amounts of data with decreasing costs, the significantly slower process of phenotypic trait characterisation has become a bottleneck in advancing plant and agronomic science. To address this challenge, several automated non-destructive phenotyping platforms have been developed [3]. Government investment in expensive, commercial high-throughput plant phenotyping tools (e.g. [4]) has led to the development of several state-of-the-art research hubs (e.g. the National Plant Phenomics Centre (www.plant-phenomics.ac.uk) in the UK). However, shared access is required and often at a premium, while hub locations can make direct research interaction challenging. Several academic and commercial lab-based tools also have been published for characterising plants, particularly the model species *Arabidopsis thaliana* (hereafter Arabidopsis). However, the wider uptake of such tools has been hampered by the local expertise required for hardware and software development. The lack of accessible hardware and appropriate algorithms for trait extraction is now widely considered the major bottleneck in the plant phenotyping field [5]. Several online tools are available for measuring plant traits (e.g. www.plant-image-analysis.org [6]) to supplement either commercial systems or support affordable solutions (e.g. www.phenotiki.com [5]). However, many image analysis systems lack robust validation and are not well supported [6].

Developing capacity to quantify plant growth phenotypes throughout the plant life cycle is important for understanding dynamic growth traits. For example, plants grow differently during the day light–dark cycle (i.e. the diel cycle), and show significant changes in phenotypic traits such as carbon partitioning to biomass, expansion rates, and leaf movement [7–14]. Regulation of such traits is co-ordinated by internal metabolic status (e.g. carbon availability, source-sink dynamics), developmental status and organ age, and the response of signalling mechanisms (e.g. the circadian clock, plant hormones) to the external environment (e.g. light quality, temperature, the length of the photoperiod). Significant research efforts are focused on investigating and modelling these complex relationships to understand how plants optimise performance in response to a dynamic growth environment [15–19]. To date, published reports that monitor dynamic growth throughout the diel cycle have been restricted to expensive and/or custom built-hardware systems [20–23]. The most commonly used method of assessing Arabidopsis rosette growth rates is overhead (top-view) 2D image acquisition (Table 1) (although 3D systems are now becoming available [13, 17]). Following image acquisition, image processing enables the extraction of plant features from the image background, either through self-calibrated threshold analysis of colour and brightness or more complex computer-vision based methods (e.g. [5, 24]).

**Table 1:** A list of published 2D imaging systems with reported measured variables for whole Arabidopsis rosettes (top-down images)

Phenotyping system	Measured variables	Imaging sensors	Background	References
PHENOPSIS	PRA	VIS	Soil	[25]
Growscreen Fluoro	PRA, stockiness, leaf count, convex hull, chlorophyll fluorescence	VIS, FLUO	Soil	[8]
WIWAM	PRA	VIS	Soil	[26]
Lab Scanalyzer (Lemnatec)	PRA, chlorophyll content and fluorescence, leaf angle, several morphometric parameters	VIS, NIR FLUO	Soil, plates	[4, 22]
OSCILLATOR	Leaf length, leaf movement	NIR	Soil	[27]
Rosette Tracker	PRA, rosette diameter compactness, stockiness, rosette temperature	VIS, TIR	Soil	[21]
PhenoPhyte	PRA	VIS	Soil	[28]
*Not named*	PRA, rosette diameter, compactness	VIS	Soil	[29]
Leaf colour segmentation	PRA, convex hull	VIS	Soil	[30]
HPGA	PRA, leaf area, leaf count, leaf length, growth modelling analysis	FLUO	Soil	[31]
Easy leaf area	PRA	VIS	Soil	[32]
*Not named*	PRA, compactness, stockiness	VIS, NIR	Black (agar plates)	[20]
PlantScreen (PSI)	PRA, chlorophyll fluorescence, several morphometric parameters	VIS, FLUO	Soil	[33]
MSU-PID	pipeline for leaf segmentation, leaf counting and leaf tracking	Depth, VIS, NIR, FLUO	Black (plants in soil)	[34]
Phenovator	PRA, chlorophyll content and fluorescence	VIS (filtered), FLUO	Black (hydroponics)	[23]
Phenotiki	PRA, leaf length, rosette diameter, compactness, stockiness, leaf count	VIS	Soil	[5]
RosettR	PRA	VIS	White (agar plates)	[24]

Commercial system providers are shown in parenthesis

*VIS* visual spectrum, *NIR* near*-*infrared, *FLUO* fluorescence, *PRA* projected rosette area

Here, we have developed an affordable image capture system (ICS) based on the Phenotiki approach [5] for the high-throughput phenotyping of soil-grown Arabidopsis plants throughout the diel cycle. The hardware is based on commonly available parts that are straightforward to order, and assembly requires some basic soldering and tools available at most fabrication facilities. The associated software module (iDIEL Plant) retains a high degree of versatility by allowing users to choose suitable parameters for experimental analysis rather than requiring adherence to a specific growth condition. To test the capacity of the ICS to capture growth data, and iDIEL Plant to identify and accurately measure growth for different plant phenotypes, we first performed a robust validation exercise and then tracked the growth of wild-type (WT) plants and two Rubisco-deficient mutants (*1a2b* and *1a3b*) that differed in growth rate and leaf colour (i.e. chlorophyll content) [35]. Our results demonstrated that this system offers a robust, low-cost solution for the uninterrupted capture of plant growth data throughout the diel cycle.

## Methods

### Construction of the image capture system (ICS)

**Fig. 1:**
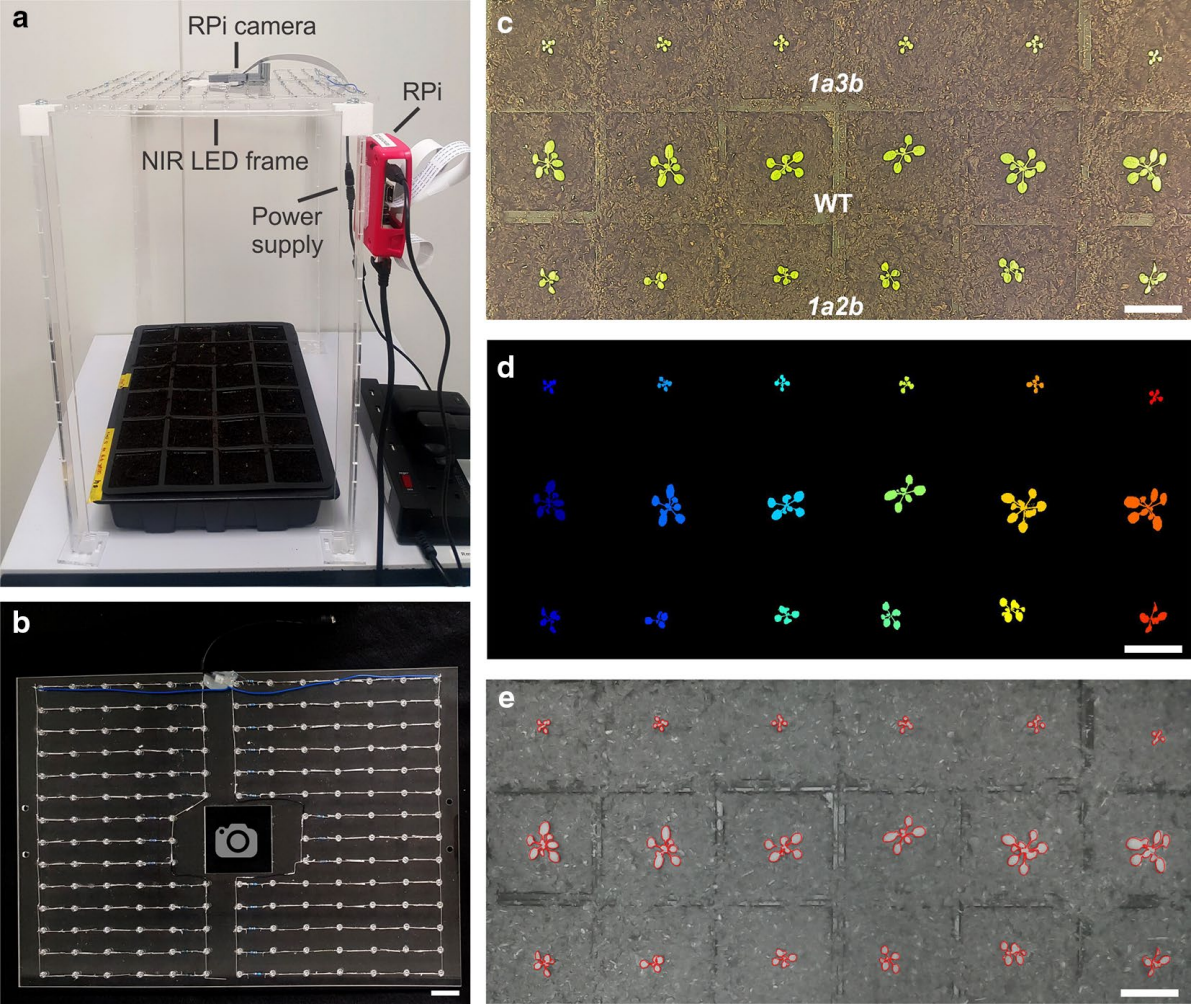
Setup of the image capture system. **a** The image capture system (ICS) consisted of a NIR LED frame, a Raspberry Pi (RPi) computer and a RPi camera (PI NoIR). **b** The NIR LED frame consisted of 173 NIR LEDS arranged in parallel circuits. The design allowed the camera to be positioned centrally in the frame (see Additional file 1 for assembly guidelines). **c** Arabidopsis WT plants and Rubisco mutants *1a3b* and *1a2b* (18 DAG) under visible light (VIS) conditions captured by the ICS with the NIR LEDs off. **d** Automated segmentation of the plants shown in (**c**). **e** Near-infrared (NIR) image of plants shown in (**c**) taken in the dark illuminated by NIR LEDs. The last image of the 18th light period (**c**) was used as a starting mask for the following dark period image (**e**). The outline of the active contour mask is shown in red. Bars: *B* = 40 mm; *C*, *D*, *E* = 25 mm

The near infra-red (NIR) LED frame was designed in qCAD [www.qcad.org]) and fabricated using a 4 mm thick clear acrylic sheet [400 × 280 mm (*L* × *W*)] (Fig. 1a, b). A total of 173 NIR 1.3 V through-hole 5 mm LEDs with a peak emittance of 940 nm [Kingbright L-7113F3C, RS (www.uk.rs-online.com)] were mounted on the acrylic sheet through paired circular holes (1.4 mm in diameter, 2.6 mm apart) using an Epilog 60 W CO2 Laser Cutter (www.epiloglaser.co.uk). The NIR LEDs were positioned 30 mm apart along the length and 20 mm apart along the width of the acrylic sheet and were soldered together using solder wire (0.5 mm, 60/40 tin/lead) and a standard soldering iron with a fine point tip. A total of 28 axial fixed resistors were soldered in line with each parallel NIR LED circuit (see Additional file 1 for further details). The soldered wires were attached to the acrylic sheet using acrylic glue. The NIR LED circuit was powered via a 12 V DC 2A power supply adaptor. We designed four supporting legs [two perpendicular acrylic strips, 420 × 20 × 4 mm (*L* × *W* × *B*), glued together] and attached an acrylic foot base [40 × 40 × 4 mm (*L* × *W* × *B*)] to each leg (Epilog 60 W CO_2_ Laser Cutter). The legs were attached to the NIR LED frame using 3D printed corner braces [Wanhao Duplicator 4 (www.wanhao3dprinter.eu)]. The uniformity of NIR illumination provided by the rig and light transmittance through the acrylic frame were measured using a UV–Vis–NIR spectrometer [USB2000 + UV–Vis (www.oceanoptics.com)].

Images were acquired using a Raspberry Pi (RPi) camera module (RPi NoIR with a 5 MP OmniVision OV5647 sensor and IR filter removed) connected to a model 2B RPi computer (www.raspberrypi.org). The RPi NoIR was mounted centrally in the acrylic frame of the rig above the plant tray (Fig. 1a) and was operated and configured remotely using the RPi Cam-Web-Interface (www.elinux.org/RPi-Cam-Web-Interface) via a web browser on a Windows computer. The images were transferred remotely using Filezilla (www.filezilla-project.org/). The total weight of the ICS was approximately 400 g.

### Plant materials and growth conditions

Initial validation of the ICS was made using Arabidopsis [*Arabidopsis thaliana* (L.) Heynh. Col-0] WT plants 15 days after germination (DAG) grown at 21 °C, ambient CO_2_, 70% relative humidity and 150 μmol photons m^−2^ s^−1^ in a 12: 12 h light: dark cycle. To further test the ICS, the growth phenotypes of Arabidopsis WT plants and two Arabidopsis Rubisco small subunit mutant lines, *1a2b* and *1a3b* (Col-0 background) [35], were compared. All plants in the latter study were grown from seeds of the same age and storage history, and were harvested from plants grown in the same environmental conditions to have a robust comparison of the three different genotypes. The seeds were sown in pots containing an organic compost-soil mix [F2 + S, Levington^®^ (www.icl-sf.com)], stratified for 3 days at 4 °C, transferred to a side-lit growth cabinet [Snijders Scientific model ECD01E, (www.snijderslabs.com)] and grown in the same conditions described above. Seedlings (8 DAG) were transplanted to individual pots in a plant tray (each pot 50 × 50 mm) containing F2 + S. The transplanted plants were maintained in the same growth cabinet conditions.

### Data acquisition

During growth experiments, images were captured every 20 min throughout the diel cycle for 16 days (9–24 DAG). The NIR LED array was synchronised with the growth cabinet to turn on at the beginning of the dark period and turn off and the beginning of the next light period. The ICS and the plant tray were not moved throughout the experiment to preserve background consistency.

### Segmentation of VIS and NIR images

For image analysis we developed a software module called iDIEL Plant. iDIEL Plant can segment Arabidopsis rosettes from both VIS (light) and NIR (dark) images and can extract quantitative estimates of projected rosette area (PRA). The software automatically numbers plants from left to right as they appear in the image, or manually by clicking on the centre of each plant. Thus, plants could be analysed regardless of arrangement in the image. ImageJ [v1.5n (imagej.nih.gov/ij)] was used to validate VIS and NIR image analyses. We further validated iDIEL Plant by examining segmentation accuracy from a published dataset of VIS and NIR Arabidopsis images [34]. The iDIEL Plant module will be made available for free download.

For growth experiments, each light and dark period contained 35 images, which were batch analysed. Plants remained separated (i.e. rosette leaves of different plants did not touch or overlap) during the growth period. Segmentation of rosettes from VIS images was done by thresholding each rosette from the background (Fig. 1c, d). iDIEL Plant offers the flexibility of choosing between three different colour spaces (RGB, HSV and Lab) and grayscale Otsu [36] (i.e., only luminance) for thresholding, which increases capacity for finding a suitable colour space for any given image dataset. Each colour space is composed of three channels: RGB is composed of red, green and blue; HSV is composed of hue, saturation (S) and value (V); and Lab is composed of lightness, a (representing green/red) and b (representing blue/yellow) channels [37, 38]. Thresholding can be done by modifying the range of each channel in a colour space until the plants remain in the foreground and the background is segmented away. The effects of the modifications can be seen in real time to give a better indication of the effectiveness of segmentation. The VIS images in this study were segmented in the HSV colour space as it provided the best segmentation results for our setup.

NIR images of the plant tray contain information saturated on just the grayscale spectrum, preventing the use of HSV colour segmentation. Thus, to batch process the dark image sets, each NIR image was segmented using a Chan-Vese active contour method to separate foreground (plant) from the background [39]. The Chan–Vese active contour algorithm is a region-based intensity driven model for delineating objects in images. Two regions are defined in the image by one or more curves that wrap around the chosen objects, dividing the image into foreground (inside the curve) and background (outside the curve). Each region is represented by a constant energy term, which is given by the average pixel value intensity within the region. The algorithm allows the curves to expand (or contract) to minimise the energy difference between the regions, resulting in segmentation of the chosen objects (i.e. rosettes). Since the Chan–Vese algorithm requires initial curves, the segmented rosettes from the last VIS image of each light period was taken as the initialization region of interest (ROI) mask for the active contour algorithm (Fig. 1c–e). The segmented rosettes from the following NIR image mask then was taken as the initialization mask for the subsequent NIR image for the remaining images in each set. As images were captured every 20 min, differences in plant appearance between sequential images were relatively small. The reverse approach can also be applied by using the opposite energy constraint (i.e. to contract) on the Chan-Vese formulation and using the first image of the light period as the starting mask and processing the NIR images in reverse starting from the end of the dark period. In this study both forward (day1-night-day2) and reverse (day2-night-day1) approaches were used and the data averaged.

LED placement and growth chamber characteristics can affect the quality of the NIR images.

iDIEL Plant is able to estimate and correct for uneven illumination (Fig. 1e). Firstly, grayscale pixel values are brought closer to the mean intensity values of the whole image. Secondly, a Gaussian blur is applied to the image to obtain a pixel intensity map of the lighter and darker regions. The pixel intensity values in the original image are then increased or decreased, respectively, based on the difference between the pixel intensity values of the intensity map and the mean intensity of the image map. The uneven illumination correction was used when analysing single channel grayscale images (i.e. the dark period and thresholding in the grayscale channel).

### Data processing

When segmentation was completed, the segmented image was correlated with the given plant indices and the area of each individual plant estimated. The data was exported to a Microsoft Excel file. Relative expansion rate (RER) was calculated in terms of PRA expansion according to Eq. 1 [13, 40]:

1 $$ {\text{RER}} = \left( {\ln  {\text{PRA}}1 {-} \ln  {\text{PRA}}2} \right)/ \left( {t1{-} t2} \right) $$

Daily RER was estimated using the PRAs obtained at the beginning and end of each light period. RER was also estimated over diel cycle using a sliding median window of 10 frames (i.e. approximately 3 h) [13].

## Results and discussion

### Performance of the image capture system (ICS)

The ICS consisted of a low-cost custom-built NIR LED frame and RPi-based image acquisition system. As the material costs of the rig were < £100, the ICS proved to be a relatively affordable but powerful tool for Arabidopsis phenotyping [5]. The relatively small size and weight of the ICS simplified handling and transport. Furthermore, the qCAD design is malleable—the LED frame could be modified or arrayed to the end-users requirements.

The use of a clear acrylic frame minimised reflection and shading effects when images were taken during the light cycle. No measurable decrease in photosynthetically active radiation (PAR) was observed when the rig was set up in the side-lit growth cabinet used for growth experiments in this study. When light levels were measured under the rig in a vertically lit growth chamber (Snijders Scientific model MC1000), an average decrease of 14% in PAR was observed (180–154 μmol photons m^−2^ s^−1^). The reduction in PAR could be compensated for by adjusting the growth cabinet light output. Thus, we recommend testing light intensity under the rig for top-light or glass house experiments.

**Fig. 2:**
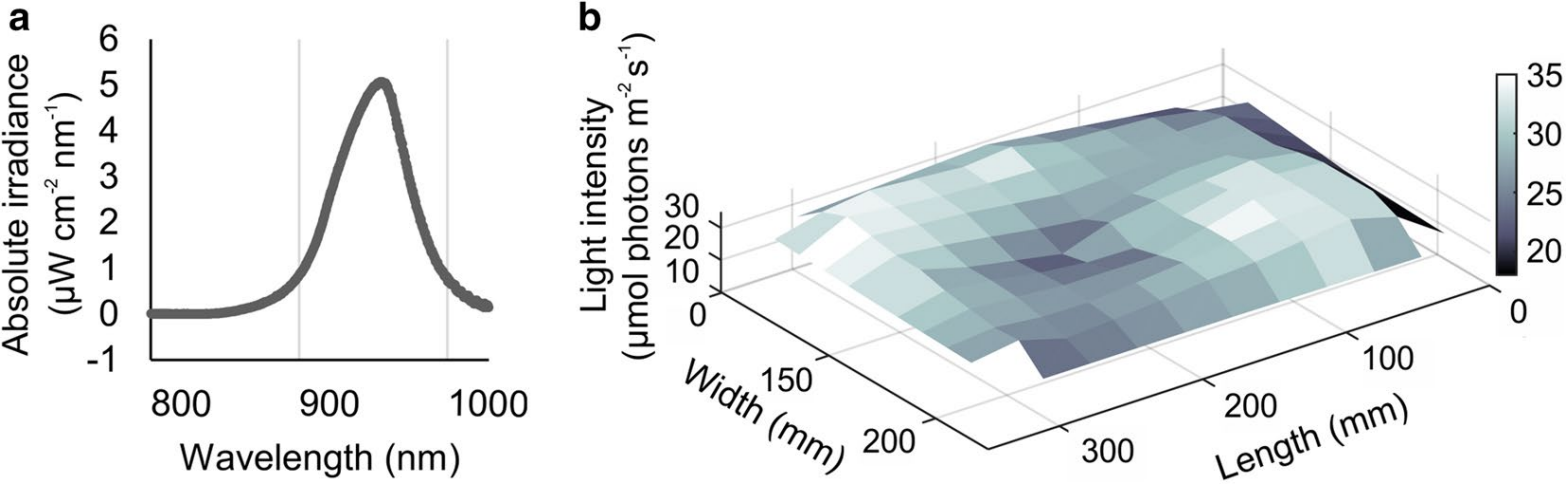
Distribution of NIR light under the ICS. **a** Emittance peak of the NIR LEDs (940 nm). **b** Light levels under the rig (420 mm beneath the NIR LED frame) in a total area of approximately 340 × 240 mm

To test the uniformity of NIR illumination we measured light quality and quantity in the area under the ICS (Fig. 2). We observed a clear peak at 940 nm with a range of 850–1000 nm, and an average intensity of 30 μmol photons m^−2^ s^−1^. A small decrease in light intensity was observed in the central area around the camera, which had a reduced density of NIR LEDS. As expected, the light intensity also showed a drop off towards the edges of the ICS frame. However, the plant tray was located well within this zone. Despite the minor variations in NIR light intensity, image capture quality was sufficient to accurately segment all plant images throughout the growth experiment.

Raspberry Pi cameras have previously been shown to produce good quality VIS images for plant trait extraction and are a low-cost and easy-to-setup alternative to expensive cameras used in other plant imaging systems [5]. Here, we found that the RPi NoIR camera was capable of capturing VIS and NIR images for image analysis. The ICS was able to acquire images at a maximum rate of ten images per minute. Furthermore, the camera could be controlled and image data transmitted remotely, giving significant flexibility to the user.

### Data processing and software validation

VIS images can be batch processed by other available software tools for plant segmentation, such as Phenotiki and Rosette Tracker [5, 21]. However, we found that automatic segmentation of NIR images for soil-grown plants was challenging using available software tools. For example, software from Zhang et al. [29] suffered from low signal-to-noise ratios and removed night images during analysis. Rosette Tracker [21] relies on corresponding chlorophyll fluorescence or VIS images for manual annotation of dark images taken with a thermal IR camera, and Dhondt et al. [20] is optimised for imaging plants grown in agar-containing Petri dishes under relatively low PAR levels (60 μmol photons m^−2^ s^−1^). Therefore, we developed a MatLab-based software module called iDIEL Plant for segmentation and improved batch extraction of Arabidopsis rosette images from both VIS and NIR images.

Processing NIR images can be difficult due to increases in background noise (e.g. soil reflectance) when using thresholding compared to VIS images. Furthermore, NIR images are grayscale and cannot use the VIS image colour spaces and channels for thresholding (Fig. 1c, e), which makes it more challenging to define contours for segmentation. To overcome this problem, we relied on an active contour algorithm that uses a VIS image obtained shortly before/after the NIR image as an initialization mask [39]. Previously, De Vylder et al. [21] developed a similar approach for the Rosette Tracker software, which uses a correspondent VIS image as a projected mask to analyse thermal infrared images (TIR). Rosette Tracker utilises a warping method that requires the user to manually identify and click on several regions in each VIS and IR corresponding image. For iDIEL Plant, image analysis was batched, such that the segmentation mask of each image was used as the initialization mask for the subsequent image. We found that this approach reduced background noise and provided a better initialisation point for the active contour algorithm.

The iDIEL Plant module currently extracts PRA estimates from VIS and NIR images, and could export segmentation masks and write raw and processed data in the database format required by Phenotiki [5]. This permits the use of the Phenotiki software to extract additional growth traits (e.g. compactness, stockiness) and utilisation of other modules within Phenotiki (e.g. leaf counting and semi-automated leaf segmentation) [41].

**Fig. 3:**
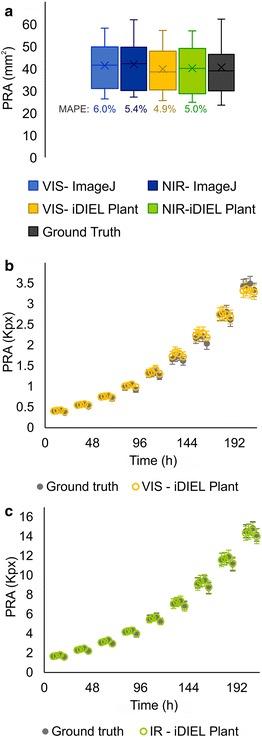
Validation of projected rosette area estimates. **a** Average projected rosette area (PRA) estimates obtained from seven individual plants (15 DAG). VIS and NIR images were analysed using iDIEL Plant (as described in the “Methods” section) or ImageJ (manual measurement). The rosettes were then detached and individual leaves were scanned and measured manually to obtain a ground truth measurement. No significant differences in PRA were observed (one-way ANOVA; p ≤ 0.05). The mean absolute percentage error (MAPE) was calculated for each dataset in relation to ground truth. **b**, **c** Validation using the Arabidopsis image dataset provided by Cruz et al. [34]. Segmentation masks for seven WT plants (plant 1, 3, 5, 7, 8, 10 and 15) [34] from VIS and NIR images at 9, 12,16 and 20 h for 9 days (15–24 DAG) are given in total number of pixels (kilopixels, Kpx) (grey). PRA estimates from respective raw VIS (yellow) and NIR (green) images analysed by iDIEL Plant are shown. Bars represent the mean ± SE. No significant differences were observed at any time point (paired Student’s *t* test; p ≤ 0.05)

Validation using ground truth measurements and/or published datasets is now considered a key requirement for developing plant imaging systems [6]. The accuracy of iDIEL Plant was initially validated by comparing PRA estimates with two different manual measurement methods (Fig. 3a). Firstly, 2D image data (VIS and NIR) were manually contoured in ImageJ to calculate rosette areas. Secondly, we used a destructive (ground truth) approach, where whole rosettes were dissected and individual leaves of each plant were scanned. The destructive method excluded the presence of features that might cause discrepancies in the estimated area obtained by 2D imaging methods, such as leaf curvature, hyponasty, hidden petioles or overlapped leaves. iDIEL Plant was able to measure PRA with high accuracy for VIS and NIR images, showing estimates close to those obtained by manual segmentation and ground truth measurements. On average, the estimates from ground truth measurements were closer to those obtained from iDIEL Plant than those from ImageJ. The manual PRA estimates from ImageJ were not significantly different from values given by iDIEL Plant. To further validate the capacity of iDIEL Plant to accurately extract PRA estimates from available datasets, we used VIS and NIR plant images provided by Cruz et al. [34] (Fig. 3b, c). The dataset contained images of Arabidopsis Col-0 WT plants grown in a 16: 8 h light: dark cycle from 15 to 24 DAG on soil (i.e. a black background). Cruz et al. [34] provided raw images and rosette segmentation masks at a temporal resolution of four images per day. The masks were used as a representative proxy for PRA and were compared with the PRA estimates obtained from iDIEL Plant following analysis of the raw images. The raw VIS images and NIR images differed in resolutions and were not co-registered, thus we analysed them independently. VIS image datasets were batch processed in iDIEL Plant using thresholding in the HSV colour space. For processing NIR images, we manually calibrated the first image of each plant in the dataset (15 DAG) using grayscale Otsu thresholding [36], and then batch processed further images using the Chan-Vese active contour approach (see “Methods” section). Comparison between the provided masks and PRA estimates produced by iDIEL Plant showed no significant difference for both VIS and IR image datasets. These validation results also demonstrated that iDIEL Plant can accurately predict PRA from images obtained using different parameters from those used in our study.

Furthermore, we tested the segmentation performance of iDIEL Plant with different soil backgrounds (Additional file 2). Comparison of the PRAs of plants placed on four common soil mixture components (F2 + S, F2 + S mixed with perlite, coco peat, and vermiculite) showed that the software could accurately segment rosettes in VIS and NIR images with a variety of different backgrounds. However, we advise against using components with reflective particles, such as perlite of vermiculite, as these can sometimes be included in segmentation if touching the rosette, potentially leading to an artificial increase in PRA. If these are required for an experiment, we recommend using a black felt fabric cover or a layer of a homogeneous dark top soil to maximize segmentation accuracy.

### Diel analyses of plant growth traits

**Fig. 4:**
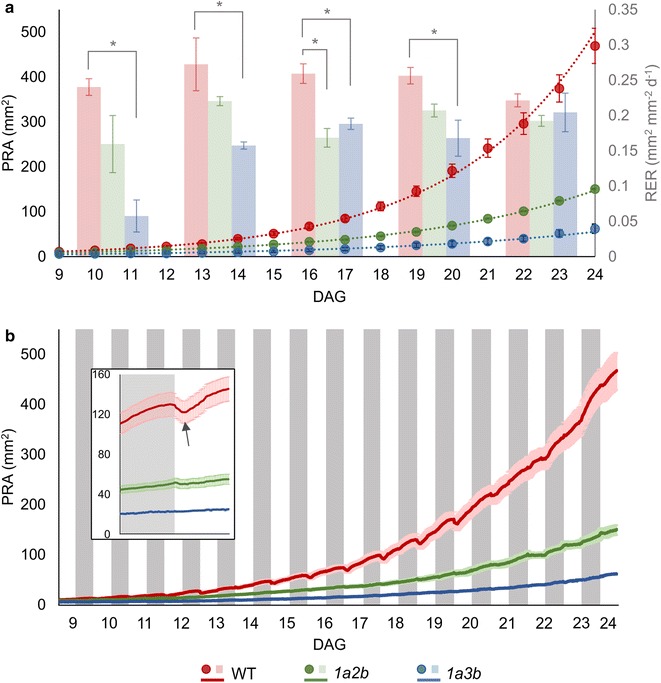
Growth analysis of Arabidopsis WT and the Rubisco mutants using iDIEL Plant. **a** Daily PRAs (represented by the dots and trend line) and relative expansion rates (RER; bars) were calculated using the last VIS image obtained in each light period over 15 days. Values are the mean ± SE of measurements made on six individual rosettes for each genotype. The RER values shown are the average RER obtained every 3 days; asterisks indicate significant difference (p ≤ 0.05) as determined by one-way ANOVA and post hoc Tukey’s multiple comparison test. **b** PRAs obtained every 20 min over the entire diel cycle. The solid darker lines represent PRA values and the lighter colours behind the lines are the mean ± SE. The grey bars represent the dark periods. The insert highlights epinastic leaf movements observed for WT plants (transition from 18th dark period to 19th light period). Leaf movements are indicated by the consistent drop in PRA (arrow) in the beginning of the light period (see Additional file 3 for video data)

Despite the reduced growth and pale leaf phenotypes of the two Rubisco mutants, iDIEL Plant successfully segmented and analysed *1a2b* and *1a3b* plants during the growth period tested. Following segmentation and trait extraction, we initially compared the end of day PRA for each genotype (Fig. 4a). WT plants showed a typical exponential growth pattern, with a PRA of 470 mm^2^ at 24 DAG. Both *1a2b* and *1a3b* showed a significantly decreased PRA compared to the WT plants throughout the growth experiment. At 24 DAG, *1a2b* had a PRA of 150 mm^2^, while *1a3b* was eightfold smaller than WT plants, reaching only 62 mm^2^. The reduced growth rates observed for *1a2b* and *1a3b* were in accordance with previous reports [35, 42].

Tracking PRA estimates at an increased temporal resolution (i.e. 20 min intervals) revealed additional growth details (Fig. 4b). We captured evidence of rhythmic leaf movements, characterised by the elevation (hyponasty) and lowering (epinasty) of the leaves during the diel cycle [11]. Leaf movement was evident for WT plants by depressions in the growth curve at the beginning of the light period (i.e. the PRA of the 2D rosette images was reduced). Video analysis revealed that WT leaves moved downwards before moving upwards again during this period (Additional file 3). Our observations correlate well with the temporal leaf movement patterns seen in other studies with WT Arabidopsis plants [11, 13]. In contrast, similar leaf movement patterns were much reduced for *1a2b* and not detected for *1a3b* at the onset of the light period. This observation may indicate that leaf movement is inhibited for both Rubisco mutants. Alternatively, leaf movements may have been less pronounced compared to WT plants and were not detected using the ICS. Previous work has shown that leaves of the starchless *pgm* mutant have reduced and delayed hyponastic responses, suggesting that changes in carbon metabolism may impact on the rhythmicity of leaf movements [13]. Thus, the Rubisco mutants used in the present study may be similarly affected by reduced carbon availability.

**Fig. 5:**
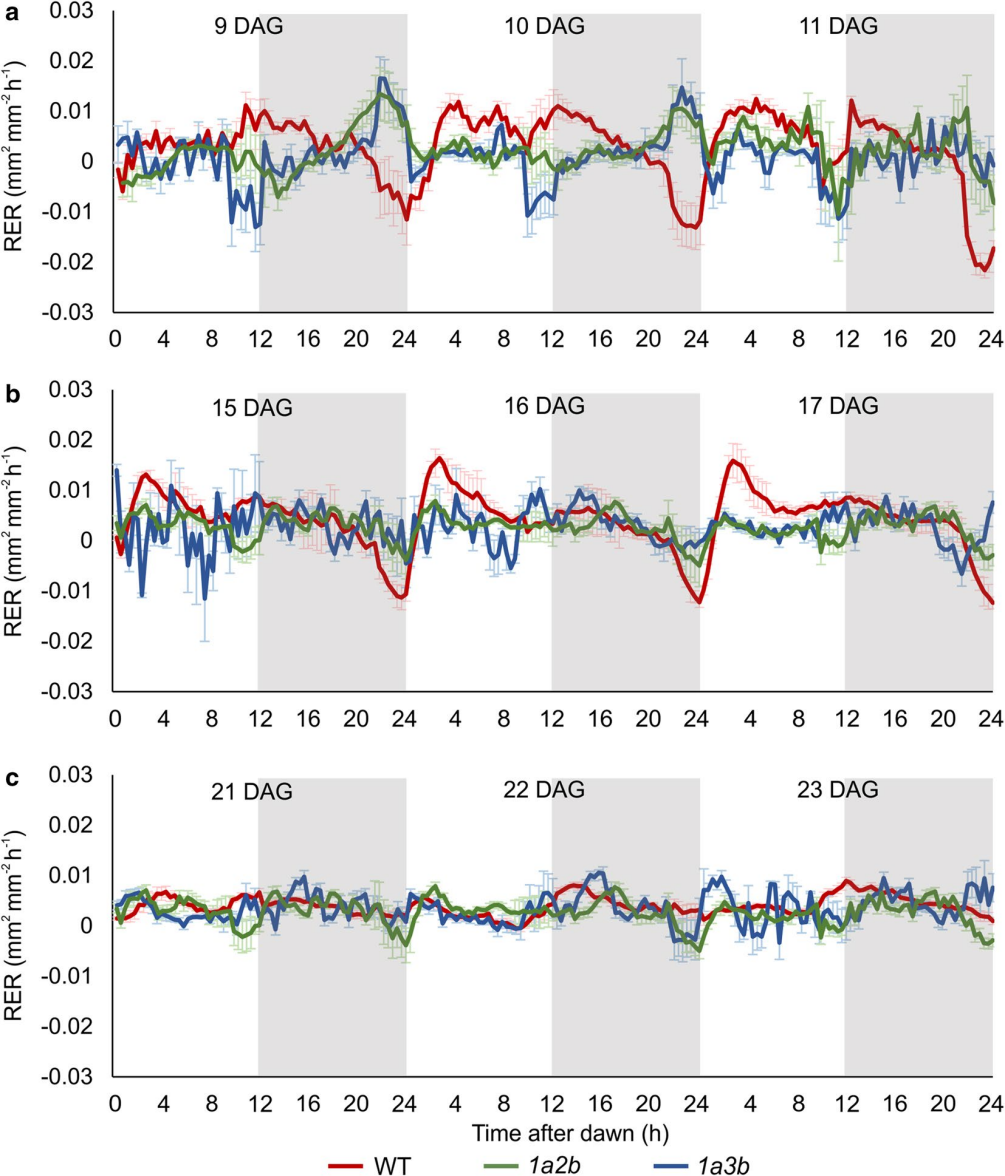
Relative expansion rate (RER) of WT and Rubisco mutants over the diel cycle during three developmental stages. The RER was estimated during three different periods of growth: **a** the young phase (9–11 DAG), **b** the maturing phase (15–17 DAG), and **c** the mature phase (21–23 DAG). The grey bars represent the dark periods

Next, we examined the 3-day average RER of each genotype over the growth period to investigate if the observed growth impairment in the Rubisco mutants was a result of a sustained reduction in RER or a decrease in RER during a particular developmental phase (Fig. 4a). WT plants had an increased RER compared to both mutants from 9 to 20 DAG. The RER of *1a3b* was significantly lower than WT plants throughout this period. In contrast, *1a2b* was on average lower than WT plants, but the observed reduction was not consistently significant. These dissimilarities in RER could be explained by the relative differences in Rubisco content for *1a2b* and *1a3b,* which are reduced by 50 and 30%, respectively [35, 42]. Rubisco content in C3 leaves is usually found in excess to facilitate dynamic adjustment to changes in the environment, such as increased irradiance or temperature. Even when Rubisco activity is reduced by up to 50%, plants may not show a significant change in growth phenotype under non-limiting growth conditions (i.e. the light levels used in this study were non-saturating) [41]. Nevertheless, both Rubisco mutants showed a progressive improvement in RER as they matured and were more similar to WT values at 21 DAG (Figs. 4a, 5), indicating that the observed slow growth phenotypes of *1a2b* and *1a3b* was primarily due to a reduced RER in the early principal growth stages of rosette development [44].

The RER of each genotype was further investigated over the diel cycle at a higher temporal resolution during three different growth phases (Fig. 5). WT plants showed a relatively consistent RER pattern at all three growth phases, which included a rise in RER during the light period followed by a decline, and then a brief peak during the beginning of the dark period followed by a steady decrease until the following light period. RER fluctuations were much less pronounced in older WT plants. These growth rate patterns were similar to those observed for Arabidopsis rosettes in previous 2D studies and high precision 3D analyses of RER in diel cycles [13, 17, 20]. Both Rubisco mutants also showed an increase in RER during the light period, but the peak was much more discrete than WT plants, particularly for *1a3b*. This observation is in accordance with the lower daily RER observed for these plants from 9 to 11 DAG (Fig. 4a). Surprisingly, younger Rubisco mutants showed an increase in RER during the dark period, typically with a higher peak than in the light period (Fig. 5a, b). In general, both Rubisco mutants showed a similar RER pattern, the difference being *1a3b* had a comparatively reduced RER. This pattern persisted in the second growth phase (15–17 DAG), but gradually switched to a WT-like pattern during the third growth phase (21–23 DAG) (Fig. 5b, c). Previously, transgenic tobacco plants with reduced Rubisco levels were characterised by a decreased accumulation of sugars and starch during the light period compared to WT plants, but typically maintained WT-like rates of starch utilisation in the dark period, such that starch reserves in the transgenic plants were more depleted by dawn [9, 43]. The latter phenomenon was attributed to an increase in sink demand for carbon compared to WT plants [41] and may account for the RER patterns observed for the younger *1a3b* and *1a2b* mutants.

## Conclusion

Our goal was to develop a simple, robust and affordable phenotyping system capable of tracking the growth of multiple plants during development throughout the diel cycle. The use of low cost components, easy-to-source hardware, and simplified construction requirements should significantly lower the entry barrier for plant researchers and facilitate scaling to more high-throughput phenotyping analyses [5]. Our ICS design enabled the acquisition of dynamic and continuous image data, while the iDIEL Plant module could batch process image data for Arabidopsis genotypes that differed in size and hue. As a caveat, 2D imaging approaches should be considered only a proxy for 3D RER, as 2D data can convolute leaf growth, leaf movement and, in older rosettes, leaf overlap [5, 20, 21]. Furthermore, leaf tissue characteristics can vary between genotypes or in response to the growth environment, thus it is important to match 2D imaging of novel or uncharacterised genotypes with destructive analyses (e.g. fresh and dry rosette weight) to determine parameters such as specific leaf area, leaf thickness and density [16, 35]. Nevertheless, 2D RER appears to be a sensitive method for detecting relative changes in rosette shape, and could be used as a proxy to indicate differences between genotypes and/or environmental factors.

## Additional files

**Additional file 1**. Detailed schematics of the image capturing system. a) Dimensions of the acrylic sheet used to construct the NIR LED array (assembled in qCAD, [www.qcad.org]). Paired holes (1.4 mm in diameter) were positioned 2.6 mm apart to allow for mounting of standard through-hole 5 mm NIR LEDs (940 nm, Kingbright L-7113F3C, RS (www.uk.rs-online.com)]. The acrylic sheet was cut using an Epilog 60 W CO_2_ Laser Cutter (www.epiloglaser.co.uk). b) Schematic of the NIR LED circuit. The array is composed of 28 parallel rows of NIR LEDs powered by a 12 V/2A power supply. The double arrows next to the LED symbols (triangle and line) represent the direction of current flow from the cathode (positive terminal) to the anode (negative terminal). All LEDs must be soldered in the right orientation, such that the cathode of one LED (represented by a grey 1) is connected to the anode (represented by a grey 2) of the next LED. The resistor values are given in ohms (Ω).

**Additional file 2**. Projected rosette area estimations of Arabidopsis rosettes on different soil backgrounds. Three mature Arabidopsis rosettes were carefully excised and imaged on four common soil mixture components under VIS and NIR illumination. a) Example of an Arabidopsis rosette under the conditions specified. The NIR images show the outline of the active contour mask based on the segmentation of the corresponding VIS image. b) Projected rosette areas (PRAs) of rosettes on four different soil backgrounds (bars represent the mean ± SE; n = 3). No significant differences in PRA were observed (one-way ANOVA; p ≤ 0.05). However, note that for soils containing reflective particles (e.g. F2 + S + Perlite and Vermiculite) PRA can be slightly higher than more homogeneous soils, as the segmentation can sometimes include the particles that are directly under a leaf.

**Additional file 3**. Video showing growth and leaf movement for three Arabidopsis genotypes. Six plants for WT (middle) and Rubisco mutants *1a3b* (top) and *1a2b* (bottom) are shown from 18 to 19 DAG at 5 frames per second.
