# Supplementary material for: A “Do-It-Yourself” phenotyping system: measuring growth and morphology throughout the diel cycle in rosette shaped plants
Source: Plant Methods. 2017 Nov 8;13:95. doi: 10.1186/s13007-017-0247-6 (PMC5678596; doi:10.1186/s13007-017-0247-6)
Supplement: Supplementary file 1 — Additional file 1. Detailed schematics of the image capturing system. a) Dimensions of the acrylic sheet used to construct the NIR LED array (assembled in qCAD, [www.qcad.org]). Paired holes (1.4 mm in diameter) were positioned 2.6 mm apart to allow for mounting of standard through-hole 5 mm NIR LEDs (940 nm, Kingbright L-7113F3C, RS (www.uk.rs-online.com)]. The acrylic sheet was cut using an Epilog 60 W CO2 Laser Cutter (www.epiloglaser.co.uk). b) Schematic of the NIR LED circuit. The array is composed of 28 parallel rows of NIR LEDs powered by a 12 V/2A power supply. The double arrows next to the LED symbols (triangle and line) represent the direction of current flow from the cathode (positive terminal) to the anode (negative terminal). All LEDs must be soldered in the right orientation, such that the cathode of one LED (represented by a grey 1) is connected to the anode (represented by a grey 2) of the next LED. The resistor values are given in ohms (Ω). [file 13007_2017_247_MOESM1_ESM.pdf]

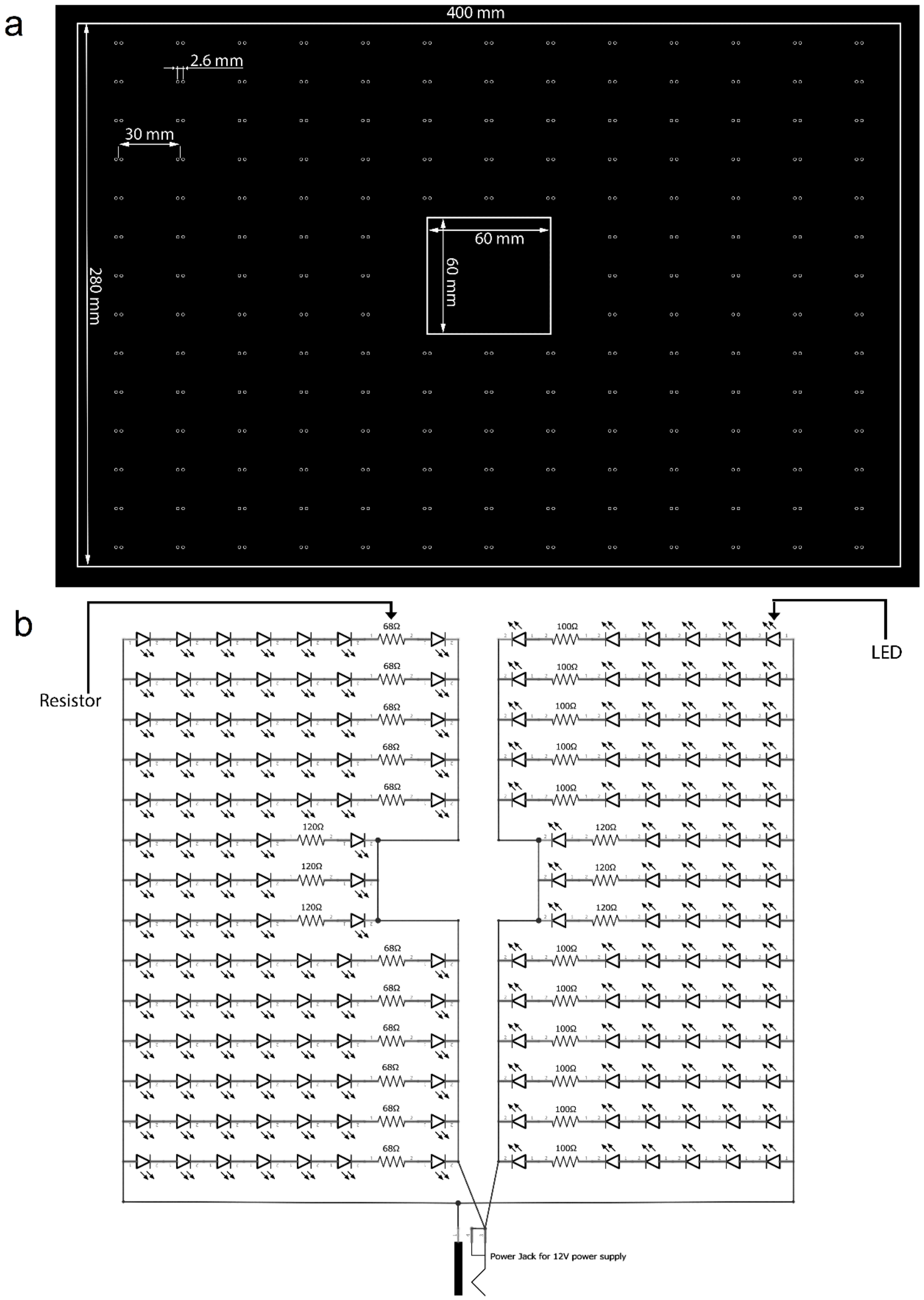

Additional File 1. Detailed schematics of the image capturing system. **a)** Dimensions of the acrylic sheet used to construct the NIR LED array (assembled in qCAD, [www.qcad.org]). Paired holes (1.4 mm in diameter) were positioned 2.6 mm apart to allow for mounting of standard through-hole 5 mm NIR LEDs (940 nm, Kingbright L-7113F3C, RS (www.uk.rs-

online.com)]. The acrylic sheet was cut using an Epilog 60W CO_2 Laser Cutter (www.epiloglaser.co.uk). **b)** Schematic of the NIR LED circuit. The array is composed of 28 parallel rows of NIR LEDs powered by a 12V/2A power supply. The double arrows next to the LED symbols (triangle and line) represent the direction of current flow from the cathode (positive terminal) to the anode (negative terminal). All LEDs must be soldered in the right orientation, such that the cathode of one LED (represented by a grey 1) is connected to the anode (represented by a grey 2) of the next LED. The resistor values are given in ohms (Ω).

Additional File 2. Video showing growth and leaf movement for three Arabidopsis genotypes. Six plants for WT (middle) and Rubisco mutants *1a3b* (top) and *1a2b* (bottom) are shown from 18 to 19 DAG at 5 frames per second.
