# Supplementary material for: A “Do-It-Yourself” phenotyping system: measuring growth and morphology throughout the diel cycle in rosette shaped plants
Source: Plant Methods. 2017 Nov 8;13:95. doi: 10.1186/s13007-017-0247-6 (PMC5678596; doi:10.1186/s13007-017-0247-6)
Supplement: Supplementary file 2 — Additional file 2. Projected rosette area estimations of Arabidopsis rosettes on different soil backgrounds. Three mature Arabidopsis rosettes were carefully excised and imaged on four common soil mixture components under VIS and NIR illumination. a) Example of an Arabidopsis rosette under the conditions specified. The NIR images show the outline of the active contour mask based on the segmentation of the corresponding VIS image. b) Projected rosette areas (PRAs) of rosettes on four different soil backgrounds (bars represent the mean ± SE; n = 3). No significant differences in PRA were observed (one-way ANOVA; p ≤ 0.05). However, note that for soils containing reflective particles (e.g. F2 + S + Perlite and Vermiculite) PRA can be slightly higher than more homogeneous soils, as the segmentation can sometimes include the particles that are directly under a leaf. [file 13007_2017_247_MOESM2_ESM.pdf]

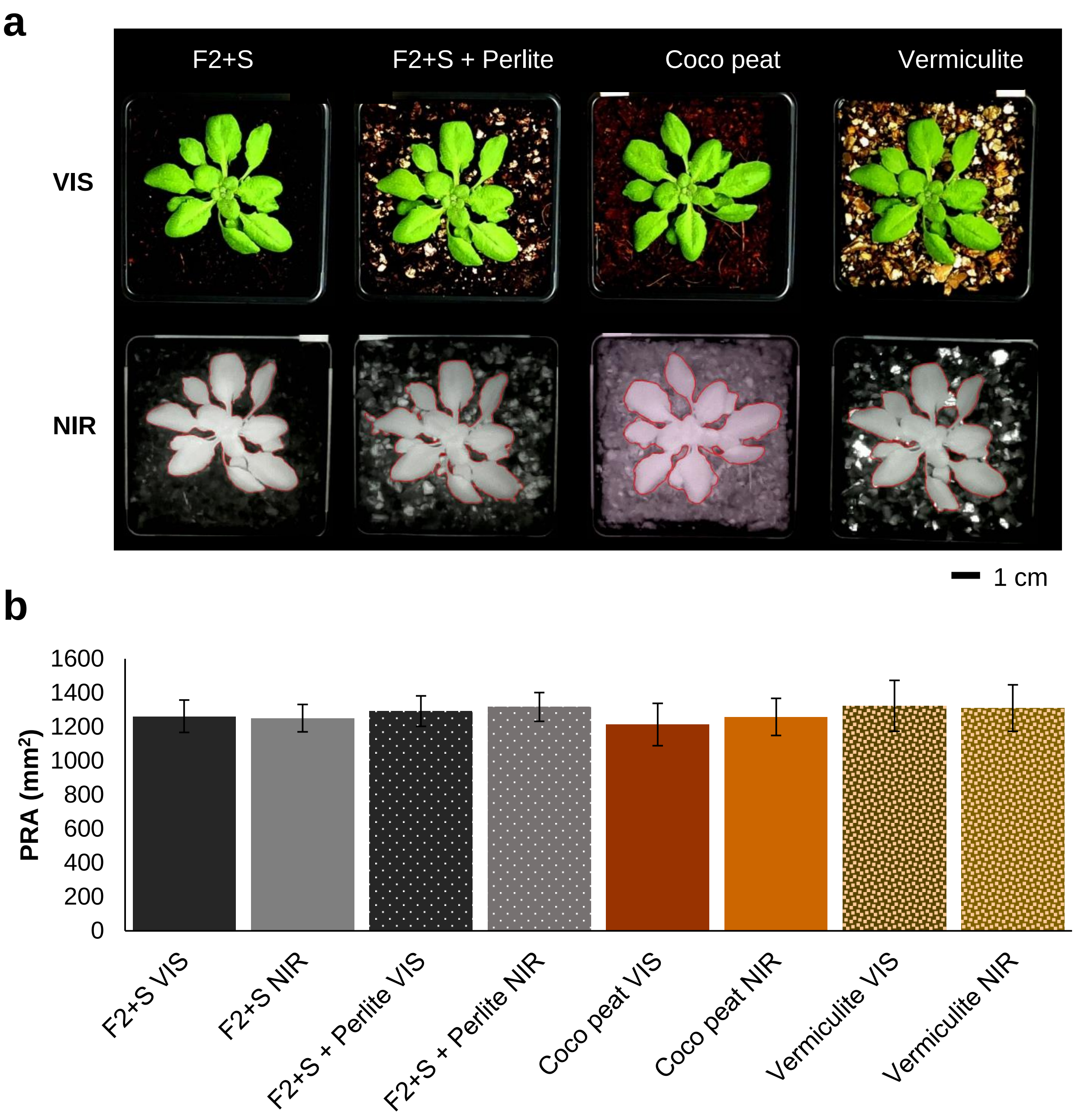

Additional File 2. Projected rosette area estimations of Arabidopsis rosettes on different soil backgrounds. Three mature Arabidopsis rosettes were carefully excised and imaged on four common soil mixture components under VIS and NIR illumination. **a)** Example of an Arabidopsis rosette under the conditions specified. The NIR images show the outline of the active contour mask based on the segmentation of the corresponding VIS image. **b)** Projected rosette areas (PRAs) of rosettes on four different soil backgrounds (bars represent the means ± SE; n=3). No significant differences in PRA were observed (one-way ANOVA; $p \leq 0.05$). However, note that for soils containing reflective particles (e.g. F2+S + Perlite and Vermiculite) PRA can be slightly higher than more homogeneous soils, as the segmentation can sometimes include the particles that are directly under a leaf.
